# Hippocampal memory enhancing activity of pine needle extract against scopolamine-induced amnesia in a mouse model

**DOI:** 10.1038/srep09651

**Published:** 2015-05-14

**Authors:** Jin-Seok Lee, Hyeong-Geug Kim, Hye-Won Lee, Jong-Min Han, Sam-Keun Lee, Dong-Woon Kim, Arthanari Saravanakumar, Chang-Gue Son

**Affiliations:** 1 Liver and Immunology Research Center, Oriental Medical College of Daejeon University, 22-5 Daehung-dong, Jung-gu, Daejeon, 301-724, Republic of Korea; 2 TKM-based Herbal Drug Research Group, Korea Institute of Oriental Medicine, Daejeon 305-811, Republic of Korea; 3 Department of Applied Chemistry, Oriental Medicine College of Daejeon University, 62, Daehak-ro, Dong-gu, Daejeon, 300-716, Republic of Korea; 4 Department of Anatomy, Brain Research Institute, Chungnam National University School of Medicine, Daejeon, Republic of Korea

## Abstract

We evaluated the neuropharmacological effects of 30% ethanolic pine needle extract (PNE) on memory impairment caused by scopolamine injection in mice hippocampus. Mice were orally pretreated with PNE (25, 50, and 100 mg/kg) or tacrine (10 mg/kg) for 7 days, and scopolamine (2 mg/kg) was injected intraperitoneally, 30 min before the Morris water maze task on first day. To evaluate memory function, the Morris water maze task was performed for 5 days consecutively. Scopolamine increased the escape latency and cumulative path-length but decreases the time spent in target quadrant, which were ameliorated by pretreatment with PNE. Oxidant-antioxidant balance, acetylcholinesterase activity, neurogenesis and their connecting pathway were abnormally altered by scopolamine in hippocampus and/or sera, while those alterations were recovered by pretreatment with PNE. As lipid peroxidation, 4HNE-positive stained cells were ameliorated in hippocampus pretreated with PNE. Pretreatment with PNE increased the proliferating cells and immature neurons against hippocampal neurogenesis suppressed by scopolamine, which was confirmed by ki67- and DCX-positive stained cells. The expression of brain-derived neurotrophic factor (BDNF) and phosphorylated cAMP response element-binding protein (pCREB) in both protein and gene were facilitated by PNE pretreatment. These findings suggest that PNE could be a potent neuropharmacological drug against amnesia, and its possible mechanism might be modulating cholinergic activity via CREB-BDNF pathway.

Alzheimer's disease and Parkinson's disease are the most common neurodegenerative disorders and a major health issue in the aging population^1^. Worldwide 33.9 million people have been diagnosed with Alzheimer's disease, and about 5.3 million people of the United States are suffering from Alzheimer's disease^2^. Neurodegenerative disorders are clinically characterized by a progressive loss of cognitive abilities, which affects learning and memory dysfunction in daily activity^3^. Neurodegenerative disorders including Alzheimer's disease results from the deposition of amyloid plaques, tau protein aggregation, cerebral oxidative stress, neuroinflammation and cholinergic dysfunction accompanied by psychological and pathophysiological complications such as anxiety, depression, concentration problems and motor disturbances^4^.

Memory impairment is attributed to dysfunction of the cholinergic system, which involves cholinergic neurons, neurotransmitters and their receptors^5^. The etiology and pathogenesis of neurodegenerative disorder remains unclear, although it is known that cholinergic dysfunction resulting from the loss of cholinergic neurons in the basal forebrain and hippocampus impairs cognitive ability^7^. Normal cholinergic activity within the central nervous system (CNS) contributes to hippocampal neurogenesis and memory improvement via the cAMP response element-binding protein/brain-derived neurotrophic factor (CREB/BDNF) pathway^8^. Thus, the primary treatment for patients with cognitive impairment is acetylcholinesterase (AChE) inhibitors such as tacrine or donepezil, which increase the availability of acetylcholine at cholinergic synapses^9^.

Oxidative stress is another well-known causative factor in the pathogenesis of neurodegenerative disorders^0^. Evidence indicates that over-production of reactive oxygen species (ROS) and reactive nitrogen species (RNS) result in damage to proteins, lipids, and nucleic acids in patients with neurodegenerative disorders^2^. Brain tissue is extremely sensitive to oxidative stress due to its high oxygen consumption, iron content, polyunsaturated fatty acids, and low antioxidant capacity^4^. Moreover, the hippocampus and amygdala are more sensitive to oxidative injury^5^, and excessive oxidative stress can lead to memory deficits by impairing hippocampal synaptic plasticity^6^.

Among numerous herbal plants, the pine needle (*Pinus densiflora* Sieb & Zucc.) is commonly used as an herbal medicine and health supplement in East-Asian countries, such as Korea and China^7^. The pine needle is utilized as an infusion of tea and supplementary health food, and is reported to be helpful in the treatment of patients with cancer, coronary heart diseases and neurodegenerative disease^9^. Despite the many uses of pine needle, there have been no studies of its neuropharmacological activities. Thus, in the present study we investigated the anti-amnesic effects of pine needle extract (PNE) on memory deficits in a mouse model of cognitive impairment caused by scopolamine.

## Results

### Compounds present in the 30% ethanolic pine needle extract

Four different kinds of flavonoids including catechin, quercetin dehydrates, astragalin, and kaempferol, when PNE sample were subjected to the HPLC. Among them, only catechin and astragalin were detected at 19.01 min and 31.34 of retention time (see Supplementary Fig. S1A and B online), and the concentration of catechin and astragalin were 0.35 ± 0.01 and 2.68 ± 0.04 respectively (see Supplementary Fig. S1C online). GC-MS data confirmed that terpenoids of α-pinene or β-pinene were not present in PNE (data not shown).

### Effect on anti-amnesia in Morris water maze task

**Figure 1:**
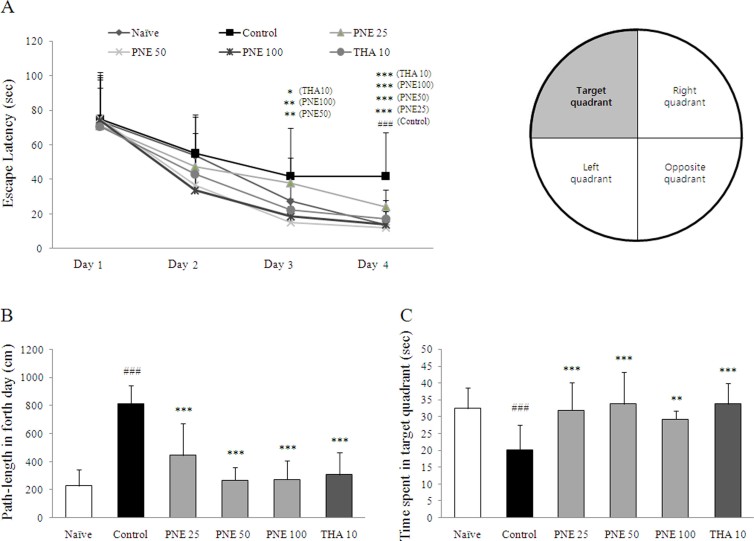
Spatial learning and memory in the Morris water maze confirm the anti-amnesic effects of PNE against scopolamine-induced memory deficits. (A) Escape latency of the 4-day acquisition trial. (B) Cumulative path-length of acquisition trial on fourth day. (C) Time spent in the target quadrant during the probe trial on day 5. Data are expressed as means ± SD (*n* = 10). ^###^*P* < 0.001 compared with the naïve group; *P < 0.05, **P < 0.01, ***P < 0.001 compared with the control group.

Scopolamine injection significantly prolonged the escape latency time and cumulative path length compared with the naïve group on fourth day of the acquisition period (approximately 3- and 4-fold respectively, *P* < 0.001). The time spent in the target quadrant was significantly shortened in the control group compared with naïve group on fifth day (*P* < 0.001). Pretreatment with PNE significantly ameliorated the escape latency (*P* < 0.001 for all groups) and the path-length (*P* < 0.001 for all groups; Fig. 1A and B) prolonged by scopolamine. On day five, PNE pretreatment groups exhibited significantly increased time spent in the target quadrant compared with the control group (*P* < 0.001 for both 25 and 50 mg/kg, *P* < 0.01 for 100 mg/kg; Fig. 1C). Tacrine had effects similar to those of PNE pretreatment.

### Effects on ROS levels in serum and hippocampus

**Figure 2:**
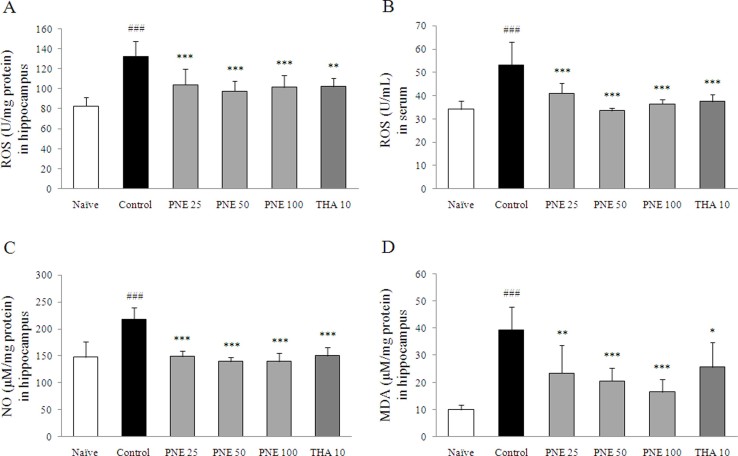
Levels of ROS, NO and MDA confirm the antioxidant effects of PNE on scopolamine-induced oxidative damage in hippocampal tissue. ROS levels in (A) the hippocampus (B) serum. (C) NO (D) MDA levels in the hippocampus. Data are expressed as means ± SD (*n* = 7). ^###^*P* < 0.001 compared with the naïve group; ***P < 0.001 compared with the control group.

Scopolamine injection significantly increased the ROS levels in both serum and hippocampal tissue compared with the naive group (approximately 1.6-fold, *P* < 0.001 in both samples). Pretreatment with PNE significantly decreased ROS levels in serum and hippocampal tissue compared with the control group (*P* < 0.001 for all groups; Fig. 2A and B); similar effects were observed in the tacrine group.

### Effects on NO and MDA level in hippocampus

NO level in hippocampal tissue was increased significantly by scopolamine injection compared with the naive group (approximately 1.5-fold, *P* < 0.001), while pretreatment with PNE significantly attenuated the increase of NO level (*P* < 0.001 for all groups; Fig. 2C).

MDA level was increased significantly by scopolamine injection in hippocampal tissue compared with the naive group (approximately 3.9-fold, *P* < 0.001), while pretreatment with PNE significantly attenuated the elevation of MDA level compared with the control group (*P* < 0.01 for 25 mg/kg, *P* < 0.001 for both 50 and 100 mg/kg; Fig. 2D). Tacrine had effects similar to those of PNE pretreatment.

### Effects on antioxidant biomarker profiles in the hippocampus

Scopolamine injection significantly depleted the antioxidant activities in hippocampal tissue, including TAC capacity (*P* < 0.001), GSH content (*P* < 0.001), activity of GSH-Rd (*P* < 0.001), GST (*P* < 0.01), SOD (*P* < 0.05), and catalase activity (*P* < 0.001), compared with the naïve group. These changes were reversed by PNE pretreatment and for all biomarkers, 50 and 100 mg/kg PNE pretreatment were more effective than 25 mg/kg PNE treatment. Interestingly, SOD activity in response to PNE pretreatment was further enhanced compared with the naïve group. In contrast, tacrine had different effects than PNE, with the exception of TAC capacity (Table 2).

### Lipid peroxidation, cell proliferation and neurogenesis in the hippocampus

**Figure 3:**
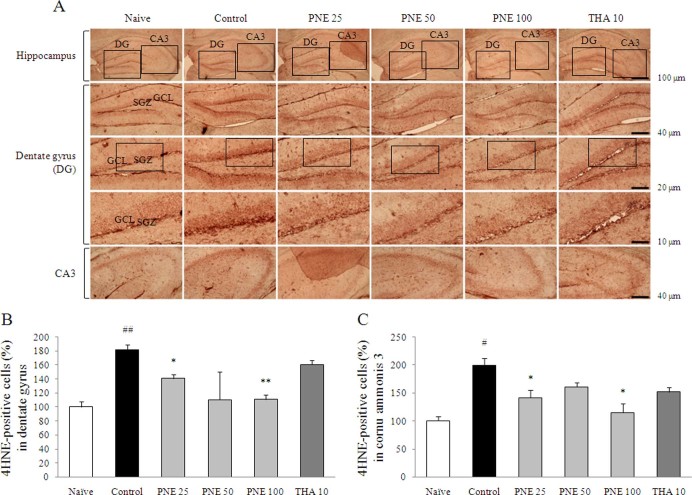
4HNE immunohistochemical analysis illustrating the inhibitory effects of PNE against scopolamine-induced lipid peroxidation in the hippocampus. (A) The strong magenta color of the 4HNE-positive stained cells in the dentate gyrus and CA3 region of the hippocampus are indicative of lipid peroxidation. Representative photomicrographs were taken at magnifications of 40, 100, 200, and 400×. Quantification of 4HNE protein adducts in (B) the dentate gyrus and (C) CA3. Data are expressed as means ± SD (*n* = 3). ^#^P < 0.05, ^##^P < 0.01 compared with the naïve group; *P < 0.05, **P < 0.01 compared with the control group. DG; dentate gyrus, CA3; cornu ammonis 3, SGZ; subgranular zone, GCL; granular cell layer.

Scopolamine injection significantly induced the lipid peroxidation in hippocampus (*P* < 0.01 in DG, *P* < 0.05 in CA3), shown as a deep red color in the dentate gyrus (DG) and cornu ammonis 3 (CA3) regions by 4HNE staining. Pretreatment with PNE significantly attenuated 4HNE-positive staining in the DG including granular cell layer (GCL), subgranular zone (SGZ) (*P* < 0.05 for 25 mg/kg, *P* < 0.01 for 100 mg/kg) and CA3 compared with the control group (*P* < 0.05 for both 25 and 100 mg/kg; Fig. 3A–C).

**Figure 4:**
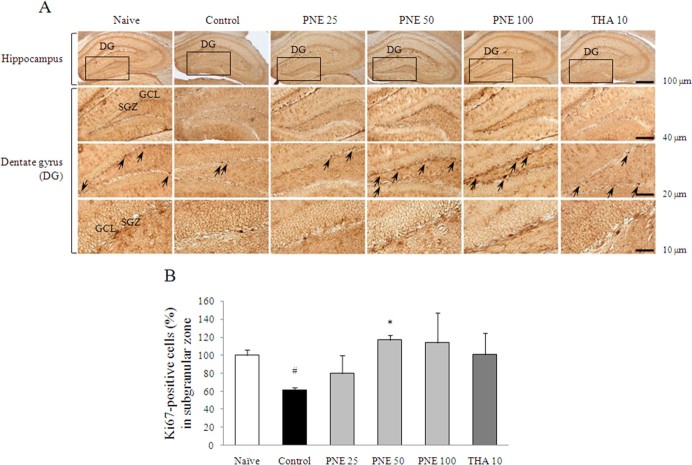
Ki67 immunohistochemical analysis of the effects of PNE on improved scopolamine-induced suppression of brain cell proliferation in the hippocampus. (A) Ki67-positive stained progenitor cell is shown as black granules in the subgranular zone of dentate gyrus. Representative photomicrographs were taken at magnifications of 40, 100, 200, and 400×. (B) Quantification of Ki67-positive cells. Data are expressed as means ± SD (*n* = 3). ^#^P < 0.05 compared with the naïve group; *P < 0.05 compared with the control group.

Scopolamine injection significantly inhibited the production of new granule cells in the hippocampal DG region, particularly in the SGZ (*P* < 0.05), as evidenced by reduced Ki67 staining. These alterations were significantly attenuated by PNE pretreatment with notable replacement of proliferating cells with Ki67-positive staining in the SGZ compared with the control group (*P* < 0.05 for 50 mg/kg; Fig. 4A and B).

**Figure 5:**
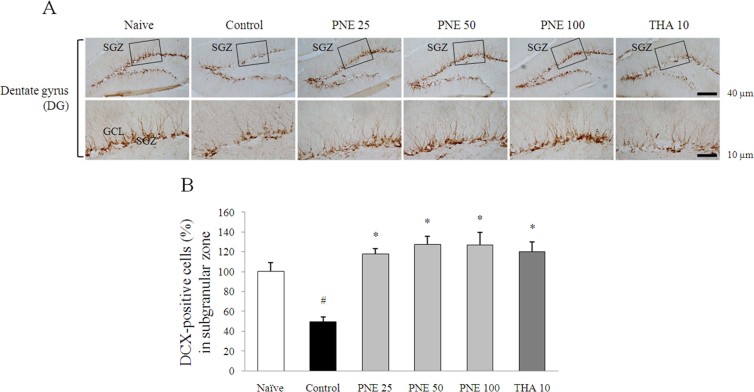
DCX immunohistochemical analysis of the effects of PNE on improved scopolamine-induced suppression of neurogenesis in the dentate gyrus. (A) DCX-positive staining in immature neurons is shown in the subgranular zone of dentate gyrus. Representative photomicrographs were taken at magnifications of 100 and 400×. (B) Quantification of DCX population. Data are expressed as means ± SD (*n* = 3). ^#^P < 0.05 compared with the naïve group; *P < 0.05, **P < 0.01 compared with the control group.

Scopolamine injection significantly suppressed the adult neurogenesis, shown as a distributed dendrites and neuron bodies in the DG region by DCX staining, particularly in the SGZ (*P* < 0.05). Pretreatment with PNE completely ameliorated the adult neurogenesis by enhancing immature neurons in the SGZ compared with the control group (*P* < 0.05 for all groups; Fig. 5A and B). In contrast, tacrine ameliorated these alterations only in DCX staining result.

### Effect on AChE activity in hippocampus

**Figure 6:**
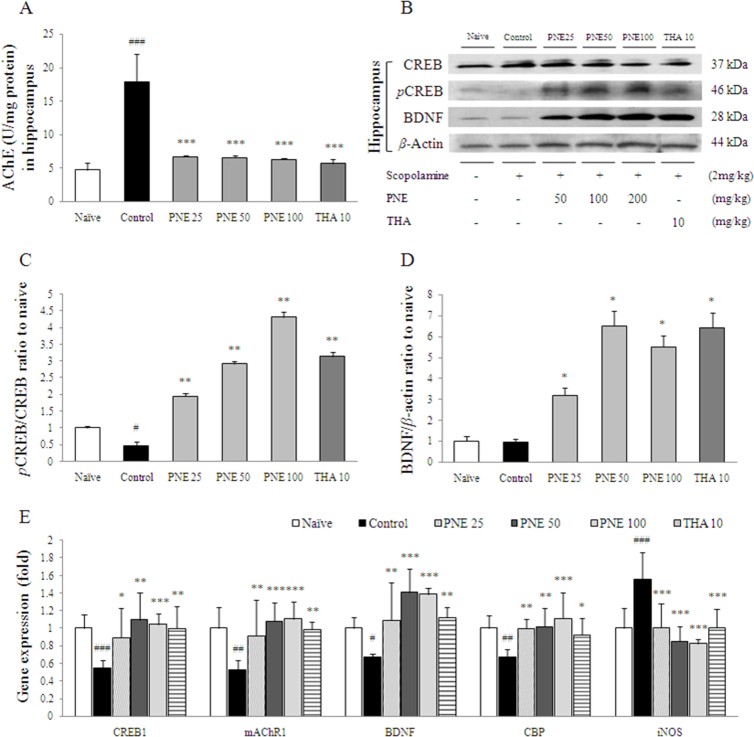
AChE activity, mAChR1, phosphorylated CREB, and BDNF protein and gene expression as possible mechanisms of PNE action. (A) AChE activity in the hippocampus (*n* = 7). (B) Phosphorylated CREB and BDNF levels in the hippocampus determined by Western blotting (*n* = 7). (C) Quantification of phosphorylated CREB/CREB intensity. (D) Quantification of BDNF/β-actin intensity. (E) Alterations in the expression of CREB1, mAChR1, BDNF, CBP and iNOS determined by real time-PCR (*n* = 6). Gene expression was normalized to that of β-actin. Data are expressed as means ± SD. ^#^*P* < 0.05, ^##^*P* < 0.01, ^###^*P* < 0.001, compared with the naïve group; *P < 0.05, **P < 0.01, ***P < 0.001 compared with the control group. PNE; pine needle extract, THA; tacrine.

AChE activity in hippocampal tissue was significantly increased by scopolamine injection compared with the naïve group (approximately 3.8-fold, *P* < 0.001), while pretreatment with PNE completely inhibited the hyper-activation of AChE compared with the control group (*P* < 0.001 for all groups; Fig. 6A). This effect was also seen with tacrine in the hippocampus.

### Western blot analysis of CREB/BDNF in the hippocampus

Scopolamine injection slightly reduced the BDNF and phospho-CREB protein levels in the hippocampus. In contrast, pretreatment with PNE increased BDNF and phospho-CREB levels compared with the control group. Tacrine had an effect on the expression of BDNF similar to that of PNE (Fig. 6B).

### Changes in mRNA levels in the hippocampus

The CREB1 (*P* < 0.001), mAChR1 (*P* < 0.01), BDNF (*P* < 0.05) and CBP (*P <* 0.01) mRNA levels were significantly down-regulated by scopolamine injection compared with the naïve group in the hippocampus. This down-regulation was ameliorated significantly by PNE pretreatment compared with the control group (*P* < 0.01 or *P* < 0.001 for all groups; Fig. 6C). Moreover, scopolamine injection significantly up-regulated the expression of iNOS in the hippocampus compared with the naïve group (approximately 1.6-fold, *P* < 0.001), while this up-regulation of iNOS was significantly ameliorated by pretreatment with PNE (*P* < 0.001 for all groups; Fig. 6C). Similar effects were observed in the tacrine group.

## Discussion

Neurodegenerative disorders are becoming a major healthcare issue as they result in progressive brain dysfunction among the aging population in industrialized countries. The major pathophysiological features involve alterations of the central cholinergic system, including cholinergic neurons, neurotransmitters and their respective receptors^0^. Several inhibitors of AChE, as well as N-methyl-D-aspartate (NMDA) receptor antagonists, are available currently for the treatment of Alzheimer's disease at mild and moderate stages, even though the mechanisms mediating these processes remain to be fully elucidated and sometimes theses drugs show adverse effects^1^. Multitarget-directed ligands and natural therapeutic agents are recently drawing attention as complementary and alternative medicine for neurodegenerative disorders^2^.

Herein, we investigated the neurotherapeutic effects of PNE on memory deficits in a mouse model of amnesia induced by scopolamine injection. To evaluate the effects of PNE on spatial learning and memory, a Morris water maze task was performed^3^. Following 4 days of acquisition training, the scopolamine treated group had prolonged escape latency (3-fold) and cumulative path-length (4-fold). This result was consistent with time spent in the target quadrant, which was reduced compared with the naïve group. As expected, pretreatment with PNE ameliorated delayed escape latency (Fig. 1A–C). These findings suggest that PNE has an anti-amnesic effect in the scopolamine-induced model.

We examined the antioxidant effects of PNE in the hippocampus since oxidative stress contributes to pathogenesis and histological changes in patients with neurodegenerative disorders^4^. The excessive production of ROS triggers neurotoxic activity through thiol- and lipid-dependent mechanisms in cell membrane^5^. The scopolamine-induced memory deficit model exhibits prominent oxidative stress and memory deficits, although the mechanism of oxidative damage in brain tissue remains unclear^7^. The hippocampus plays a crucial role in short- and long-term memory, as well as spatial memory^8^, and is highly susceptible to oxidative stress^9^. As expected, scopolamine injection induced oxidative stress in hippocampus, as evidenced by increased levels of ROS, NO and MDA. These alterations were attenuated significantly by PNE pretreatment (Fig. 2A–D). Previous studies showed that scopolamine treatment elevated the production of MDA in the cerebral cortex and hippocampus^0^. These results were consistent with changes in iNOS gene expression in the hippocampus (Fig. 6C).

To protect tissues against oxidative damage, cellular organisms maintaining an antioxidant system composed of non-enzymatic and enzymatic components. In our study, scopolamine injection significantly depleted antioxidant capacity of TAC, SOD, catalase and the GSH-redox system in the hippocampus, whereas these alterations were ameliorated significantly by pretreatment with PNE (Table 2). These results suggested that the antioxidant potential of PNE contributed to neuronal plasticity and memory function. Furthermore, in the DG and CA3 region of the hippocampus, scopolamine induced lipid peroxidation, shown as positively stained 4HNE cells in the GCL and SGZ. However, pretreatment with PNE completely attenuated the over-production of 4HNE (Fig 3). Increased 4HNE is a key histopathological feature of various diseases, including Alzheimer's disease, diabetes and cancer^1^. In the hippocampus, dentate granule cells are responsible for the separation of each episodic memory, such as object and place, and the CA3 pyramidal neurons receive and store auto-associative memories in their network^2^.

The new neurons are generated as neural progenitor cells in the SGZ of hippocampal DG, in the adult mammalian brain, including humans^3^. As well known, adult hippocampal neurogenesis plays a crucial role in hippocampal memory function^4^. Accordingly, enhancing hippocampal neurogenesis may be an efficient therapeutic target for Alzheimer's disease. We confirmed the significantly decreased proliferating cells and immature neurons by Ki67 and DCX staining of the scopolamine-induced mouse hippocampus, while pretreatment with PNE markedly ameliorated repression of reproducing cell and neuronal precursor cells in the SGZ (Fig. 4 and 5). These findings can predicts the production of new granule cells, maturation of neurons as well as hippocampal neurogenesis. The neurogenesis and gliogenesis are regulated by various molecular factors, such as neurotransmitters, neurotrophins, hormones and their respective signaling pathways^5^.

To investigate the role of PNE in neurogenesis, we examined cholinergic activity, neurotransmitter levels and subsequent signaling pathways. Scopolamine injection augmented AChE activity 4-fold in hippocampal tissue, whereas pretreatment with PNE completely attenuated the excessive activation of AChE (Fig. 6A). Maintenance of ACh levels is necessary for normal memory function, but excessive AChE activity leads to disruption of ACh in hippocampal cholinergic synapses^6^. In the synaptic cleft, ACh binds to post-synaptic mAChRs and the synaptic signal communicates sequentially to cyclic adenosine monophosphate/protein kinase A (cAMP/PKA)-CREB signaling pathway via G-coupled protein receptors^7^. In our result, the down-regulation of mAChR-1 gene expression by scopolamine in hippocampal tissues was normalized significantly by PNE pretreatment (Fig. 6C). Previous studies have revealed that patients with Alzheimer's disease have down-regulated expression of mAChR-1^8^, which correlates with memory disruption in a mAChR-1 knockout mouse model^9^.

In contrast, the transcription factor CREB is essential for memory and synaptic plasticity in the CNS. Disturbance of phosphorylated CREB within the hippocampal region leads to the progression of neurodegenerative diseases such as Alzheimer's disease, Parkinson's disease and Huntington's disease^0^. Previous studies indicated that CREB has a neuroprotective effect against ROS-mediated cell toxicity^1^, and activation of CREB ameliorated cognitive impairment via the cholinergic system^8^. In our study, hippocampal phospho-CREB was reduced by scopolamine injection, which was increased by pretreatment with PNE (Fig. 6B). The expression of CREB1 and CBP, a co-factor of CREB, supported these results (Fig. 6C). BDNF is known to improve learning ability and neurogenesis via phospho-CREB signaling, and mice deficient in BDNF and pro-BDNF exhibit an aging process characteristic of Alzheimer's disease^2^. In our study, pretreatment with PNE markedly increased BDNF protein levels (Fig. 6B), which was correlated with BDNF mRNA levels in hippocampal tissue (Fig. 6C).

*Pinus densiflora*, also called Japanese Red Pine, grows naturally in East Asian countries and has been used traditionally as a supplement in various foods and folk medicine. Previous studies well evidenced the pine needle has potent antioxidant effects against oxidative damage and apoptosis, with anti-obesity and anti-cancer effects^4^. To date, several active compounds have been isolated from organic-solvent extractions, including flavonoids, terpenoids and tannin^6^. Among them, the flavonoid-derived compounds are well-known for enhancement of memory function via stimulating neurogenesis, regulating neurotrophins, and protecting neurons against oxidative and metabolic stress^8^. In this study we observed the chemical characters of PNE via performing HPLC analysis using four flavonoid-derived compounds including catechin, astragalin, quercetin dehydrate, and kaempferol. As a result of HPLC analysis, catechin and astragalin were detected in PNE, and we further explored the quantitative analysis of those compounds (Fig. S1A–C). It was well corresponded to the previous studies that both catechin and astragalin showed potent neuroprotective effects on the various brain damage model. Catechin showed its protective effects on the severe brain oxidative damage via enhancement of antioxidant capacities as well as especially inactivation of NF-κB in pyramidal cells of hippocampal CA1 region^9^. On the other hand, the astragalin prevented brain ischemic injury through anti-apoptotic effect and decreases of neuronal cell adhesion molecules^0^.

In conclusion, our results demonstrate that PNE has an anti-amnesic effect, which could be mediated by hippocampal neurogenesis, cholinergic activity, CREB-BDNF pathway and its antioxidant properties. This suggests that the *Pinus densiflora* leaf may be a promising treatment for patients with neurodegenerative disorders. Further study should aim to confirm the neuroprotective effects and its corresponded mechanisms using active compounds such as catechin and astragalin.

## Material and Methods

### Materials

Pine needles (*Pinus densiflora* Sieb & Zucc.) were collected from Guryong-Mountain in Chungwon-gun, Chungbuk, Korea. Fifty grams of dried pine needles were extracted with 0.5 L of 30% ethanol at 25°C for 72 h, and suspension was filtered using a 300-mesh 50-mm filter paper (Advantec, Toyo Roshi Kaisah, Tokyo, Japan). Filtrate was concentrated in a rotary evaporator and lyophilized. The final yield of the extract was 10.46% w/w, and it was stored at −80°C. Fingerprinting analysis of PNE was measured by high-performance liquid chromatography (HPLC) and gas chromatography/mass spectrometry (GC/MS).

### Chemicals

The reagents were obtained from Sigma (St. Louis, MO, USA); 3,3′-diamino-benzidine (DAB), reduced glutathione (GSH) radioimmunoprecipitation assay (RIPA), scopolamine hydrobromide, skim milk, 9-Amino-1,2,3,4-tetrahydroacridine hydrochloride hydrate (tacrine), 1,1,3,3-tetraethoxypropane (TEP). The other reagents were obtained from the following vendors: CREB, phospho-CREB, BDNF, beta-actin, and secondary horseradish peroxidase (HRP)-conjugated antibodies for western blotting (Abcam, Cambridge, MA; and Santa Cruz Biotechnology; Santa Cruz, CA), doublecortin (DCX), 4-hydroxynonenal (4HNE), Ki67, biotinylated secondary antibodies, and avidin-biotin peroxidase complex (Abcam, Cambridge, MA; Santa Cruz Biotechnology, Santa Cruz, CA; and Vector, Burlingame, CA) for immunohistochemical staining, thiobarbituric acid (TBA; Lancaster Co., Lancashire, England), H_2_O_2_, (Junsei Chemical Co., Ltd., Tokyo, Japan).

### Animals

Sixty specific pathogen-free C57BL/6N male mice (12 weeks old; 24–27 g) were purchased from Koatech (Gyeonggido, Korea). They received food (Cargill Agri Furina, Gyeonggido, Korea) and water *ad libitum*, and were housed in a room maintained at 23 ± 2°C with a 12-h light-dark cycle. After acclimatization for one week, the mice were randomly divided into six groups (*n* = 10 per group): naïve, scopolamine, PNE pretreatment group (25, 50 or 100 mg/kg) and tacrine group (10 mg/kg, as a positive control). All groups were orally administered with distilled water (naïve and control), PNE, or tacrine for seven days prior to scopolamine injection. Except naïve group, scopolamine was dissolved in 0.9% physiological saline (2 mg/kg), and injected once intraperitoneally, 30 min before the Morris water maze task on first day.

The dose of PNE was determined based on prescreening results. The protocol was approved by the Institutional Animal Care and Use Committee of Daejeon University (DJUARB 2013-031), and was conducted in accordance with the Guide for the Care and Use of Laboratory Animals published by the United States National Institutes of Health (NIH).

### Morris water maze task

Morris water maze task was performed in a circular pool (100-cm diameter × 50-cm height) with a circular acrylic platform (10-cm diameter × 35-cm height). The location of platform can be discriminated by visual cue. The pool was filled with milk water (22 ± 1°C) and divided into equal quadrants. A platform was placed in one of the quadrants ~1 cm below the surface as described previously^1^. Data were recorded using a video camera connected to the corresponding software (Smart Junior, Panlab SL; Barcelona, Spain).

Mice were placed on the platform for 10 s, and removed from the pool. Mice were given acquisition trial for 4 days. The escape latency and cumulative path-length were recorded during each acquisition trial. On day 5^th^, all mice were subjected to probe trial without platform, and were recorded for 120 s. The time spent in the target quadrant was measured for spatial learning and memory.

### Sample preparation

All mice were sacrificed under ether anesthesia after 1 h following the Morris water maze. Blood was collected by the method followed by serum. Serum was collected by centrifugation at 3000 rpm for 15 min at 4°C, and the hippocampal region from the whole brain was isolated immediately, and then samples were stored at −80°C or in RNAlater (Ambion, TX, USA). In each group, three mouse brains were fixed in 4% paraformaldehyde, and hippocampi of remaining seven mice was used for biochemical analysis, western blot and real-time PCR analysis. The part of hippocampus was homogenized on ice using RIPA buffer, and other part of hippocampus was used for isolation of RNA.

### ROS levels

The levels of ROS in serum and hippocampus were determined as described previously^2^. Absorbance was measured at 505 nm using a UV spectrophotometer (Molecular Devices; Sunnyvale, CA, USA). The results were calculated using H_2_O_2_ as a standard and expressed as unit/mL.

### NO and MDA levels

The level of nitric oxide (NO) in hippocampus was determined using the Griess method^3^. The purple azo dye product was measured at 540 nm using a UV spectrophotometer.

The level of lipid peroxidation in hippocampus was determined by measuring malondialdehyde (MDA) using the thiobarbituric acid reactive substance (TBARS) method as described previously^4^. Absorbance at 535 and 520 nm was measured using a spectrophotometer. The concentration of TBARS was calculated using a standard TEP and the results were expressed as μmol/mg protein.

### TAC capacity

Total antioxidant capacity (TAC) in hippocampus was determined as described previously^5^. The level of TAC was expressed as the gallic acid equivalent antioxidant capacity (GEAC). The absorbance at 600 nm was measured using a UV spectrophotometer.

### Total GSH content, GSH-Rd and GST activities

Glutathione (GSH) content in hippocampus was determined as described previously^6^. Absorbance at 405 nm was measured using a UV spectrophotometer.

GSH-Rd activity in hippocampus was determined as described previously^7^. Absorbance at 41 m was measured using UV spectrophotometer. Enzyme activity was calculated using the following formula: enzyme activity (unit/mL) = [(Δsample – Δblank) × (dilution factor)]/[14.15 mM^–1^cm^−1^ × (volume of sample in mL)].

Glutathione S-transferase (GST) activity in hippocampus was determined using a GST assay kit (Sigma, St. Louis, MO, USA) according to the manufacturer's protocol. Absorbance at 340 nm was measured using UV spectrophotometer. Enzyme activity was calculated using the following formula: Enzyme activity: (unit/mL) = [(ΔA340)/min × 0.2 × dilution factor]/(5.3 mM^–1^ cm^−1^ × volume of enzyme sample tested).

### SOD and catalase activities

Superoxide dismutase (SOD) activity in hippocampus was determined using a SOD assay kit (Dojindo Laboratories; Kumamoto, Japan) according to the manufacturer's protocol. Absorbance was measured at 450 nm using UV spectrophotometer. Dilutions of bovine erythrocyte SOD ranging from 0.01–50 unit/mL were used as standards.

Catalase activity in hippocampus was determined as described previously^8^. Absorbance of the purple formaldehyde product at 550 nm was measured using a UV spectrophotometer.

### Immunohistochemical stain analysis

Immunohistochemical analysis was performed by assessing cell proliferation with Ki67, neurogenesis with DCX and lipid peroxidation with 4HNE staining in hippocampus. Three brain tissues of each group were immersed in the fixative solution for 4 h. The brain was cryoprotected in 30% sucrose, embedded in tissue-freezing medium with liquid nitrogen, and cut into coronal frozen sections (35 μm) using a Leica CM3050 cryostat. Sections were stored under anti-freeze buffer.

Parallel free-floating sections were subjected to endogenous peroxidase quenched with 1% H_2_O_2_ in PBS, followed by treatment with blocking buffer (5% normal chicken serum in PBS and 0.3% Triton X-100 for overnight at 4°C) and incubated with primary 4HNE (1:200, ab48506, Abcam), Ki67 (1:200, ab15580, Abcam) and DCX (1:200, sc-8066, Santa cruz biotechnology) antibodies. After washing with PBS, tissues were incubated with a biotinylated goat anti-rabbit (1:400, Vector, BA-1000), goat anti-mouse (1:400, Vector, BA-9200) and rabbit anti-goat (1:400, BA-5000, Vector) secondary antibody. The tissues were subsequently exposed to an avidin-biotin peroxidase complex (Vectastain ABC kit, Vector) for 2 h. The peroxidase activity was visualized using a stable diaminobenzidine solution. All immunoreactions were observed using an Axio-phot microscope (Carl Zeiss, Germany) and these results were quantified using the Image J 1.46 software (NIH, Bethesda, MD, USA).

### AChE activity

Acetylcholinesterase (AChE) activity in hippocampus was determined using a AChE activity assay kit (AAT Bioquest; Sunnyvale, CA, USA) according to the manufacturer's protocol. Absorbance at 410 nm was measured using a UV spectrophotometer.

### Western blot analysis

The expression of BDNF and CREB/phospho-CREB proteins in hippocampus was evaluated by Western blot. The proteins from homogenates were separated by 10% polyacrylamide gel electrophoresis and transferred to polyvinylidene fluoride (PVDF) membranes. After blocking in 5% skim milk, the membranes were probed overnight at 4°C with primary antibodies (CREB, p-CREB, BDNF and β-actin). The membranes were washed and incubated for 2 h with HRP-conjugated anti-rabbit antibody. Western blots were visualized using an enhanced chemiluminescence (ECL) advanced kit.

### Quantitative real-time PCR analysis

The mRNA expression of genes encoding CREB1, mAChR1, BDNF, CREB-binding protein (CBP), and inducible nitric oxide synthase (iNOS) in hippocampus was measured by real-time PCR. Total RNA was isolated from hippocampus using an RNeasy Mini Kit (QIAGEN, Valencia, CA, USA) and cDNA synthesized using a High-Capacity cDNA reverse transcription kit (Ambion, Austin, TX, USA). Real-time PCR was performed using SYBRGreen PCR Master Mix (Applied Biosystems; Foster City, CA, USA) and PCR amplification was performed using a standard protocol with the IQ5 PCR Thermal Cycler (Bio-Rad, Hercules, CA, USA). Information of primers was summarized in Table 1.

### Statistical analysis

All data are expressed as mean ± standard deviation (SD). The statistical significance differences between the groups were evaluated by one-way analysis of variance (ANOVA) followed by *post hoc* multiple comparisons with Fisher's LSD t-test using the IBM SPSS statistics software, ver. 20.0 (SPSS Inc., Chicago, IL, USA). Differences at *P* < 0.05, *P* < 0.01, or *P* < 0.001 were considered statistically significant.

## Author Contributions

J.S. Lee (Jin-Seok Lee) wrote the main manuscript text, and conducted experiments. H.G. Kim (Hyeong-Geug Kim) prepared the figure 3A–C and 6B–D, and extracted the pine needle. H.W. Lee (Hye-Won Lee) prepared the Supplementary figure 1 (HPLC analysis). J.H. Han (Jong-Min Han) supported the behavioral test for Morris water maze, and reviewed the describing manuscript. S.K. Lee (Sam-Keun Lee) performed the GC-MS analysis. D.W. Kim (Dong-Woon Kim) prepared the figure 5. Arthanari Saravanakumar checked the English grammar. C.G. Son (Chang-Gue Son) supervised the manuscript, and directed final version of all contents. All authors reviewed and approved this manuscript.

## Supplementary Material

Supplementary Information Supplementary information

## Figures and Tables

**Table 1:** Sequence of the primers used in real-time PCR analysis

Gene (number)	Primer sequencing (Forward and Reverse)	Product size (base pair)	Annealing temperature (°C)
CREB 1 (NM_013497)	5′-ACA GTG CCA ACC CCC ATT TA-3′ 5′-GTA CCC CAT CCG TAC CAT TGT T-3′	100	59
mAChR 1 (NM_001112697)	5′-AGT GGC ATT CAT CGG GAT CA-3′ 5′-CTT GAG CTC TGT GTT GAC CTT GA-3′	100	60
BDNF (NM_001048139)	5′-CAC TTT TGA GCA CGT CAT CGA A-3′ 5′-CAC CCG GGA AGT GTA CAA GTC-3′	104	60
CBP (NM_001025432)	5′-CTG GCA GAC CTC GGA AAG AA-3′ 5′-CTG GCG CCG CAA AAA CT-3′	100	59
iNOS (NM_010927)	5′-GGC AGC CTG TGA GAC CTT TG-3′ 5′-TGC ATT GGA AGT GAA GCG TTT-3′	120	60
β-actin (NM_007393)	5′-GGC ACC ACA CCT TCT ACA ATG A-3′ 5′-ATC TTT TCA CGG TTG GCC TTA G-3′	100	59

CREB; cAMP response element-binding protein, mAChR; muscarinic acetylcholine receptor, BDNF; brain-derived neurotrophic factor, CBP; CREB-binding protein, iNOS; inducible nitric oxide synthase, β-actin as a housekeeping gene.

**Table 2:** Effects on biomarkers of antioxidant in hippocampus

Treatment	Naive	Control	PNE (mg/kg)			Tacrine (mg/kg)
			25	50	100	10
TAC (μM/mg protein)	59.86 ± 10.34	40.82 ± 5.87###	56.88 ± 3.81***	58.17 ± 5.81***	57.58 ± 6.68***	55.96 ± 3.95***
GSH (μM/mg protein)	11.20 ± 0.97	7.24 ± 1.69###	9.69 ± 0.69***	10.47 ± 1.17***	9.80 ± 0.71***	8.20 ± 0.84
GSH-Rd (U/mg protein)	4.62 ± 0.33	3.47 ± 0.48###	3.78 ± 0.68	4.16 ± 0.29**	4.07 ± 0.51*	3.58 ± 0.25
GST (U/mg protein)	0.37 ± 0.06	0.29 ± 0.03##	0.30 ± 0.02	0.37 ± 0.08**	0.38 ± 0.03***	0.30 ± 0.03
SOD (U/mg protein)	38.92 ± 7.11	32.30 ± 2.03#	40.98 ± 5.80*	39.59 ± 6.43*	42.69 ± 7.72**	33.55 ± 5.58
Catalase (U/mg protein)	737.43 ± 116.15	575.57 ± 32.14###	642.21 ± 68.24	664.07 ± 60.39*	685.07 ± 33.59**	610.46 ±56.44

Data are expressed as the mean ± SD (*n* = 7). ^#^*P* < 0.05, ^##^*P* < 0.01, ^###^*P* < 0.001 compared with the naïve group; *P < 0.05, **P < 0.01, ***P < 0.001 compared with the control group.

TAC, total antioxidant capacity; GSH, glutathione; GSH-Rd, glutathione reductase; GST. glutathione S-transferase; SOD, superoxide dismutase.
