# Supplementary material for: Hippocampal memory enhancing activity of pine needle extract against scopolamine-induced amnesia in a mouse model
Source: Sci Rep. 2015 May 14;5:9651. doi: 10.1038/srep09651 (PMC4431316; doi:10.1038/srep09651)
Supplement: Supplementary Information [file srep09651-s1.doc]

**Hippocampal memory enhancing activity of pine needle in scopolamine-induced mouse model**

Jin-Seok Lee ^a^, Hyeong-Geug Kim ^a^, Hye-Won Lee ^b^, Jong-Min Han ^a^, Sam-Keun Lee ^c^, Dong-Woon Kim ^d^, Arthanari Saravanakumar ^a^, Chang-Gue Son ^a, *^

*^a^ Liver and Immunology Research Center, Oriental Medical Collage of Daejeon University, 22-5 Daehung-dong, Jung-gu, Daejeon, 301-724, Republic of Korea*

*^b^* *TKM-based Herbal Drug Research Group, Korea Institute of Oriental Medicine, Daejeon 305-811, Republic of Korea*

*^c^ Department of Applied Chemistry, Oriental Medicine Collage of Daejeon University, 62, Daehak-ro, Dong-gu, Daejeon, 300-716, Republic of Korea*

*^d^ Department of Anatomy, Brain Research Institute, Chungnam National University School of Medicine, Daejeon, Republic of Korea*

**Supplementary information**

**Fingerprinting.** To obtain the fingerprinting analysis the lyophilized PNE sample was dissolved in 50 % of methanol (20 mg/mL), and four different kinds of flavonoids solutions of catechin, astragalin, quercetin dehydrate, kaempferol (250 μg/mL in methanol) were prepared in 50% methanol and stored at 4 °C. The above solutions were filtered through an Acrodisc® LC 13 mm Syringe filter (0.45 μm pore size, Ann Arbor, MI). Firstly to obtain the quantitative analysis of PNE and reference compounds, the above stock solutions were performed to the HPLC. After observed the containing compounds in PNE, the qualificative analysis was done. The two of compounds such as catechin and astragalin were prepared by six concentrations of diluted solutions with methanol. All of calibration curves were attained by assessing the peak areas for six concentrations in the range of 0.31 to 500 μg/mL for each sample. The linearity of the peak area (y) versus concentration (*x*, μg/mL) curve for each component was used to calculate the contents in PNE. The quantitative analysis was performed under the simultaneous conditions using an 1100 HPLC (Agilent Technologies, Santa Clara, CA) equipped with an autosampler (G11313A), column oven (GA1316A), binary pump (G1312), diode-array-detector (DAD), and degasser (GA1379A). The analytical column with a Gemini C18 (4.6×250 mm; particle size 5 μm; Phenomenex, Torrance, CA) and was kept at 30 °C during performance. The data were acquired and processed by chemstation software (Agilent Technologies, Santa Clara, CA). The mobile phase conditions contained 10% acetonitrile in water with 0.05% formic acid (A) and 90% acetonitrile in water (B). The gradient flow was as follows; 0 - 10 min, 5-10% B; 10 - 15 min, 10-20% B; 15 - 30 min, 20-25% B; 30 - 40 min, 25-60% B; 40 - 50 min, 60-100%. The analysis was operated at a flow rate of 1.0 mL/min, and detected at 280 nm. The injection volume was 10 μL.

For additional analysis of the two putative terpenoids α-pinene and β-pinene in PNE, gas chromatography-mass spectrometry (GC-MS) (Agilent Technologies, Santa Clara, CA, USA) was performed. PNE (100 mg) was suspended in 3-mL diethyl ether and 2.5 µL of this solution was injected into a GC-MSD. GC was carried out using Agilent 7890A equipment with a 30-m × 250-µm × 0.25-µm capillary column. The initial column temperature of 40°C was held for 1 min and increased to 300°C at 8°C/min. The injector, detector and interface temperatures were maintained at 250°C. GC-MS was analyzed using Agilent 5975C mass spectrometer with column. Electron impact mass spectra were recorded at 70 eV. Identification was performed on the basis of peak enhancement by comparison with the NIST 5.0 mass spectra library.

**Supplementary Figure 1.**


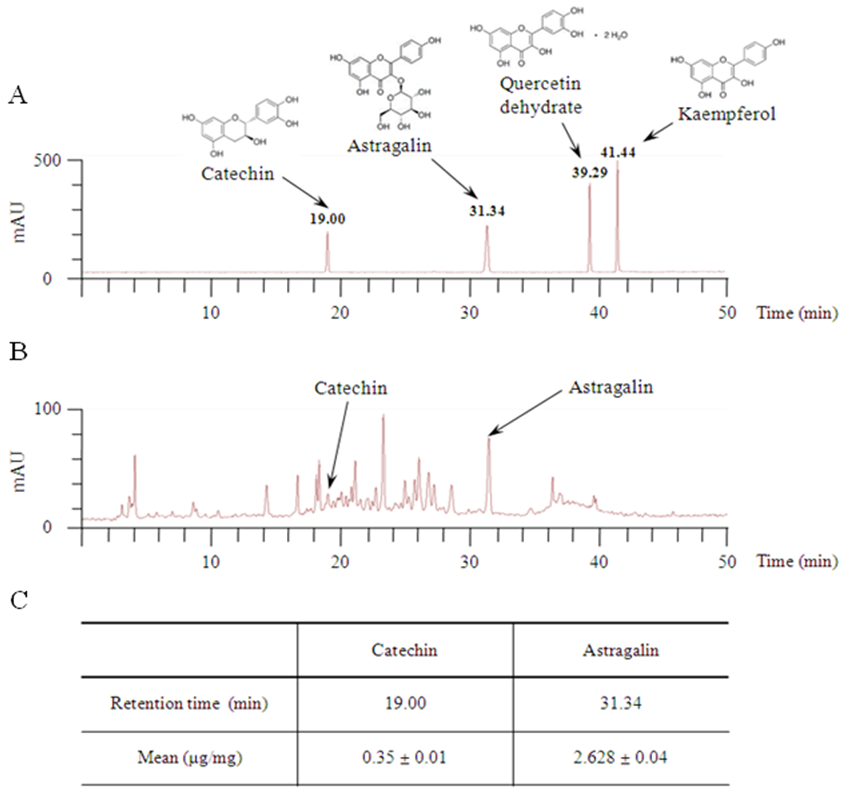


**Supplementary figure 1.** HPLC chromatograms of 30% ethanol pine needle extract (PNE). (A) A HPLC analysis of the four reference compounds was conducted. (B) PNE was subjected to HPLC analysis. (C) Retention time and quantification of main two compounds.

**Figure 6B full-length gel and blots**


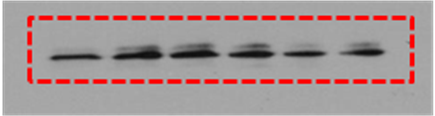
 CREB (37 kDa)


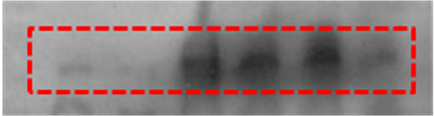
 p-CREB (46 kDa)


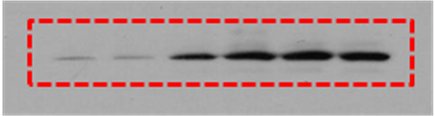
 BDNF (28 kDa)


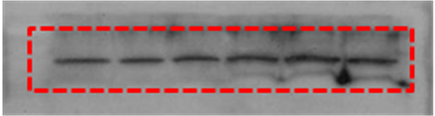
 β-actin (44 kDa)
